# FANS Unfixed: Isolation and Proteomic Analysis of Mouse Cell Type-Specific Brain Nuclei

**DOI:** 10.1021/acs.jproteome.4c00161

**Published:** 2024-07-26

**Authors:** Lucy Bedwell, Myrto Mavrotas, Nikita Demchenko, Reuben M. Yaa, Brittannie Willis, Zuzana Demianova, Nelofer Syed, Harry J. Whitwell, Alexi Nott

**Affiliations:** † Department of Brain Sciences, Imperial College London, London W12 0NN, U.K.; ‡ UK Dementia Research Institute, Imperial College London, London W12 0NN, U.K.; § MRC Laboratory of Medical Sciences, Du Cane Road, London W12 0NN, U.K.; ∥ Department of Metabolism, Digestion, and Reproduction, Imperial College London, London W12 0NN, U.K.; ⊥ PreOmics GmbH, D-82152 Martinsried, Germany

**Keywords:** epigenetics, proteomics, cell-type-specific, microglia, neurons, oligodendrocytes, FANS

## Abstract

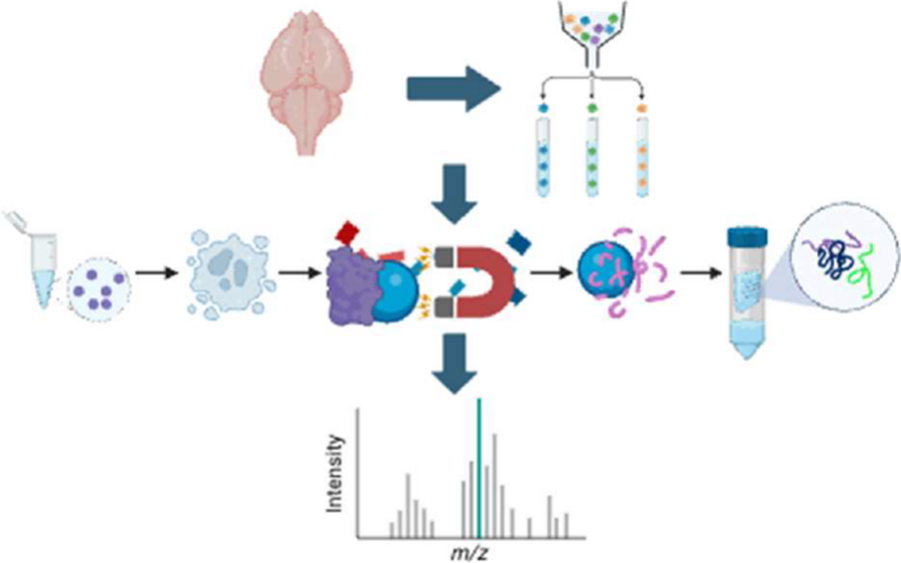

Epigenetic-mediated gene regulation orchestrates brain cell-type gene expression programs, and epigenetic dysregulation is a major driver of aging and disease-associated changes. Proteins that mediate gene regulation are mostly localized to the nucleus; however, nuclear-localized proteins are often underrepresented in gene expression studies and have been understudied in the context of the brain. To address this challenge, we have optimized an approach for nuclei isolation that is compatible with proteomic analysis. This was coupled to a mass spectrometry protocol for detecting proteins in low-concentration samples. We have generated nuclear proteomes for neurons, microglia, and oligodendrocytes from the mouse brain cortex and identified cell-type nuclear proteins associated with chromatin structure and organization, chromatin modifiers such as transcription factors, and RNA-binding proteins, among others. Our nuclear proteomics platform paves the way for assessing brain cell type changes in the nuclear proteome across health and disease, such as neurodevelopmental, aging, neurodegenerative, and neuroinflammatory conditions. Data are available via ProteomeXchange with the identifier PXD053515.

## Introduction

Brain-related conditions are associated with perturbations across multiple cell types. For example, in neurodegenerative disorders such as Alzheimer’s disease (AD), hallmarks include synaptic loss, neuronal death, loss of white matter, astrogliosis, as well as neuroinflammation.^1,2^ Single-cell gene expression studies have identified dysregulated AD-associated cell-type gene expression programs that correlate with these pathologies.^3−8^ Homeostatic and disease-associated gene expression programs are established through epigenomic chromatin states that are initiated, maintained, and read by chromatin regulators, such as transcription factors.^9^ Chromatin regulators bind to gene regulatory regions, such as enhancers and promoters, to augment the expression of nearby genes.^10^ These regulatory elements are known to integrate intra- and extra-cellular signals, resulting in context-specific transcriptional outputs.^11^ Examining the protein expression of chromatin regulators is critical to infer how environmental signals guide cellular identity and responses, and how this is perturbed in disease. Understanding how disease-associated phenotypic changes in the cell state are regulated at the proteome level is an important step in the modulation of these states and potentially influencing disease course.

Analysis of bulk brain tissue masks disease-specific changes that occur in low-abundance cell types. Microglia, the resident immune cells of the brain, have been implicated in the cellular response of Alzheimer’s disease.^12−14^ Furthermore, (epi)genetic and gene expression studies have suggested a genetic underpinning of microglia in Alzheimer’s disease.^15−23^ However, microglia account for 5–10% of all brain cells,^24^ and have been underrepresented in bulk tissue analysis.

Isolating cells from the brain requires fresh brain tissue, which is not readily available for human archived samples and can be technically challenging for cell types with complex morphologies, such as neurons. In addition, the correlation between gene expression and protein levels is often limited. For example, *STAT1* and *TBX21* transcription factors have equivalent mRNA levels in T immune cells, however, STAT1 protein expression is 150 times higher.^25,25^ Other transcription factors, such as NF-κB, are expressed during homeostasis but require signal-dependent translocation from the cytoplasm to the nucleus to function,^26^ which cannot be detected by gene expression analysis. Nuclei can be isolated from frozen tissue according to the cell type of origin, allowing for the analysis of nuclear-localized chromatin factors from archival samples. However, little is known about the nuclear proteome of brain cell types, and how changes in chromatin-associated proteins, such as transcription factors and chromatin remodelers, drive cellular phenotypes in response to environmental perturbations and disease.

To examine the nuclear proteome of brain cell types, we coupled fluorescence-activated nuclei sorting (FANS) with proteomic mass-spectrometry analysis. We established an unfixed nuclei isolation method for the major brain cell types from frozen tissue for downstream protein extraction and mass spectrometry. This method has been optimized for FANS-enriched rare cell type-specific samples using low input material in large collection volumes. We tested multiple protein extraction approaches and found that the single-pot, solid phase-enhanced sample preparation with In-StageTip Sample Preparation (SP3-iST) (PreOmics) enabled robust nuclear lysis, protein extraction, and digestion, providing reliable peptide purification. Our approach can be used to identify proteins from rare cell types and/or organelles that are collected in high volumes with low protein concentrations and can be used to study physiological and disease-associated conditions in the brain.

## Methods

### Animals

Fresh frozen brains from adult (∼8 weeks) female C57BL/6J mice were sourced from Charles River UK, Ltd. Unless otherwise stated, the brains were provided as two separate hemispheres, with the cerebellum removed and discarded. Brains were stored at −80 °C upon arrival until processing for nuclei isolation, followed by proteomics. Bulk mouse brain proteomics (whole cell lysates) was performed on separate brain samples obtained from fresh-frozen C57BL/6 mice aged 6–8 weeks that were sectioned at 10 μm thickness, thaw-mounted onto glass slides and stored at −20 °C.

### Nuclei Isolation

Unfixed nuclei isolation was performed using a modified protocol from.^27^ Frozen mouse cortices were thawed for 5 min on ice in 5 mL lysis buffer (0.32 M Sucrose, 5 mM CaCl_2_, 3 mM Mg-Acetate, 1 mM EDTA, 10 mM Tris-HCl pH 8 and 0.1% Triton), with 150 μL protease inhibitor (Protease Inhibitor Cocktail (100X), Thermo Scientific, USA) and 150 μL of 0.1 M DTT (DTT was made fresh and added to a stock of lysis buffer on the day of extraction). Cortices were homogenized by douncing 12–15 strokes with a loose pestle followed by 10 strokes with a tight pestle (Wheaton Dounce Tissue Grinder, 7 mL). The 5 mL homogenate was strained through a 70 μm filter into a 50 mL tube and a further 5 mL lysis buffer was used to wash the dounce and passed through the 70 μm filter (10 mL total). The homogenate was passed through a 40 μm filter, using 5 mL of lysis buffer to wash out the previous tube (15 mL total). The sample was centrifuged for 10 min, 600×*g* at 4 °C (Heraeus Megafuge 16R, Thermo Scientific) with a swinging bucket rotor (TX-400, Thermo Scientific). The nuclei pellet was washed with 5 mL wash buffer (0.32 M sucrose, 5 mM CaCl_2_, 3 mM Mg acetate, 1 mM EDTA, 10 mM Tris-HCl, pH 8, and Protease Inhibitor Cocktail (1:100)) and centrifuged for 10 min, 600×*g* at 4 °C. Three washes were completed in total. A final wash was performed with FANS buffer (1% Bovine serum albumin, 1 mM EDTA in 50 mL phosphate buffer saline (PBS), and Protease Inhibitor Cocktail (1:100)). Nuclei were resuspended in 0.6 mL FANS buffer and 1% of the sample (6 μL) was added to 0.6 mL FANS buffer for the unstained control.

Samples were incubated with phycoerythrin (PE)-conjugated rabbit PU.1 antibody (1:100, Cell Signaling Technologies, 81886S) overnight at 4 °C. The next day, the samples were incubated with Alexa Fluor 647-conjugated Olig2 (1:2500, Abcam, ab225100 [EPR2673]) and Alexa Fluor 488-conjugated NeuN (1:2500, MilliporeSigma, MAB377X [A60]) for 1.5 h. Nuclei were washed in DPBS supplemented with 1 mM EDTA and centrifuged for 10 min, 600×*g* at 4 °C. The stained nuclei pellet was resuspended in 0.5 mL DPBS with 1 mM EDTA and passed through a 30 μm FACS cell-strainer. The sample volume was increased to 3–4 mL with DPBS, 1 mM EDTA and Protease Inhibitor Cocktail (1:100). Unstained controls were resuspended in 0.3 mL DPBS and 1 mM EDTA and passed through 30 μm FACS cell strainers.

Fixed nuclei were isolated using our established protocol^28^ and were used to compare with FANS plots of unfixed nuclei.

### Fluorescent-Activated Nuclei Sorting (FANs)

Sorting was performed on a BD FACSAria at the Medical Research Council (MRC) London Institute of Medical Sciences (LMS) Flow Cytometry Facility, Imperial College London. Before sorting, 1 mg mL^–1^ 4′, 6-diamidino-2-phenylindole, dihydrochloride (DAPI; DNA stain) (1:500) was added to the samples for 20 min. Unstained and DAPI-only controls were analyzed to validate the gating strategy. Forward versus side scatter was used to identify nuclei based on size and doublets were excluded. Plots of NeuN-488 versus PU.1-PE, and NeuN-488 versus Olig2–647 were used to identify the target populations. Sample holders were cooled to 5 °C, and 200,000 nuclei were sorted into Protein LoBind 1.5 mL tubes (Eppendorf) containing 300 μL Sorting Buffer (1 mM EDTA in DPBS). After sorting, samples were processed for mass spectrometry using either the freeze–thaw lysis, urea lysis, or SP3-iST lysis protocols (PreOmics), as described below.

### Freeze–Thaw Lysis

FANS nuclei were snap-frozen without removal of the supernatant (volume ∼1–1.5 mL) and stored at −80 °C. Samples were thawed at room temperature and then heated at 90 °C for 10 min. Sample volumes were reduced (<150 μL) using an Eppendorf Concentrator plus. The sample volumes were adjusted to 200 μL with 40 mM CAA (2-Chloroacetamide 98.0%, Sigma-Aldrich), 10 mM TCEP (Thermo Scientific), 50 mM TEAB (Triethylammonium bicarbonate buffer, pH 8.5, Sigma-Aldrich) and ddH_2_O. Nuclei were repeat heated to 90 °C for 5 min and left to cool to room temperature. Trypsin (Promega, stock 0.1 μg μL^–1^) was added at 1:100 dilution to the samples and digested overnight at 37 °C.

### Urea Lysis

FANS nuclei were pelleted at 600×*g* for 15 min at 4 °C. Supernatant was either removed entirely or 50 μL was left for lyophilization. Pelleted or lyophilized samples were resuspended in 50 μL PBS and 50 μL 8 M urea (final concentration 4 M urea). Samples were centrifuged at 500×*g* for 5 min and incubated at room temperature for 10 min. Samples were sonicated in a water bath for 10 min and centrifuged at 10,000×*g* for 10 min at 4 °C. Sample volumes were adjusted to 200 μL with 40 mM CAA, 10 mM TCEP, 50 mM TEAB and ddH_2_O.

### SP3-iST Lysis

FANS nuclei were kept on ice and Triton-X added at 0.5% (V/V) and centrifuged at 600×*g* at 4 °C for 15 min. The supernatant was reduced to 500 μL and samples were snap-frozen on dry ice and stored at −80 °C. SP3 beads (PreOmics) were prepared according to the manufacturer’s instructions, 250 μL of SP3 LYSE buffer was added, and nuclei samples were incubated at 95 °C for 10 min at 1200 rpm (rpm) (Eppendorf ThermoMixer). Samples were cooled to room temperature, and nuclei were sonicated to shear DNA (10 cycles, 30 s on/off). 750 μL SP3 BIND was added to the samples with 20 μL of prepared SP3 magnetic beads and incubated at room temperature for 60 min at 1200 rpm to facilitate bead-binding. Bound proteins were washed using a magnetic separator with three rounds of 150 μL SP3 WASH buffer. An on-bead digest step was performed with 25 μL iST-LYSE (2-FOLD) and 25 μL of resuspended DIGEST added to the beads. The samples were incubated for 1 h at 37 °C at 1200 rpm. 100 μL of the STOP reagent was added and the samples were cooled to room temperature and transferred to an iST cartridge. Cartridges were spun at 3800×*g* to remove excess liquid. Samples were washed with WASH 1 and WASH 2 reagents and centrifuged at 3800×*g* for 3 min or until all liquid flowed through. The iST cartridges were transferred to clean 1.5 mL collection tubes. Samples were eluted using 100 μL of ELUTE buffer and centrifugation at 3800×*g*. Elution was repeated twice (200 μL final volume). Eluted peptides were dried completely using an Eppendorf Concentrator plus at 45 °C for 2–3 h. Dried peptides were snap-frozen and stored at −80 °C.

### FANS-Proteomics

Peptides were dissolved in 10 μL LC-MS buffer (0.1% V/V formic acid (FA)) supplemented with 25 fmol μL^–1^ enolase digest (Waters) by sonicating for 10 min in a water bath before centrifuging at 17,000×*g* for 10 min. Samples were transferred to glass insert-containing LC vials (Waters, Massachusetts) from which 8 μL was injected into the LC-MS system.

Nuclei were analyzed by nanoLC-MS^E^ using an Acquity UPLC M-Class ultraperformance liquid chromatography system (Waters, Massachusetts) coupled to a Synapt G2S mass spectrometer (Waters, Massachusetts). Peptides were injected into the trap column (nanoEase M/Z Symmetry C18 100um X 20 mm, Waters, Massachusetts) and washed for 5 min with 95% Reagent A (0.1% FA) and 5% Reagent B (ACN, 0.1% FA) with a flow rate of 15 μL min^–1^. Separation was performed across a nanoEase M/Z HSS C18 T3 75 μM X 200 mm column (Waters, Massachusetts) with a flow rate of 0.3 μL min^–1^, with a 90 min gradient of Reagent B (5–40%). The column was washed with 85% B for 10 min before equilibrating with 5% Reagent B for 14 min. Mass spectra were acquired between 50 and 2000 Da using HDMSe (resolution mode and positive mode). Scan time lasted 0.5 s using a ramped collision energy (19–45 V).

Data were normalized and peaked in Progenesis QI for Proteomics (Linear Dynamics) with default settings before database searching using the Ion Accounting algorithm. Proteins were identified by examining LCMS data for peptides against a Swiss-Prot Human database (canonical sequences downloaded 18/11/2021) supplemented with yeast Enolase (Uniprot accession P00924). Cysteine carbamidomethylation was used as a fixed modification of proteins, whereas methionine oxidation was variable. Up to two missed cleavages were permitted. The false discovery rate threshold was set at 4%. Proteins were quantified with the top three quantitation (Hi3) relative to the peak intensities of the three most frequent peptides.

### Bulk Mouse Brain Proteomics (Whole Cell Lysate)

A 5 × 5 mm section of mouse brain was carefully scraped off a 10 μm cryosection, and protein was extracted. Twenty μg protein was digested using the SP4 method with minor changes.^29^ Reduction and alkylation were performed with 5 mM TCEP and 20 mM chloracetamide for 45 min at 37 °C before the addition of acetonitrile. The remaining procedure was performed with glass beads as described by Johnston et al. 2022.^29^ For LC-MS/MS analysis, samples were dissolved in 0.1% formic acid at 0.1 μg μL^–1^. Peptides were separated by HPLC (M-Class, Waters) across a 2.6 μm Kinetex XB-C18 100 Column, 150 × 0.3 mm (Phenomenex) using a 20 min linear gradient (3–30% acetonitrile, 0.1% formic acid) at a flow rate of 10 μL min^–1^ and a column temperature of 30 °C. Peptides were analyzed by ZenoSWATH on a 7600 QTOF (Sciex) configured with 85 variable windows spanning 400–900 *m*/*z* and a 0.1 s accumulation time. Over the range of 140–1750 *m*/*z*, fragment ions were collected with an accumulation time of 13 ms. Data were collected in positive polarity with a 5000 V ionization voltage, a source temperature of 200 °C, ion source gases of 20 and 60 psi, and a curtain gas of 35 psi. Before loading the next sample, the column was washed for 4 min with 80% acetonitrile and equilibrated for 5 min with 97% water. Raw MS files were processed with DIA-NN (version 1.8.1), with match between runs (MBR) enabled and filtered to 1% FDR. The UniProt Mouse (*Mus musculus*) proteome Swiss-Prot database (Proteome ID: UP000000589; Downloaded: 09/12/2022) was used to generate a predicted spectral library. Trypsin/P digestion was specified with two missed cleavages permitted, N-term methionine excision (fixed modification), C carbamidomethylation (fixed modification), oxidized methionine (variable modification), peptide length range of 7–30 amino acids, a precursor charge range of 1–4, fragment ion *m*/*z* range of 140–1800 and precursor *m*/*z* range of 400–1500.

### ImageStream Analysis and Nuclei Size Quantification

ImageStream analysis was performed at the MRC-LMS Flow Cytometry Facility, Imperial College London. Nuclei from neurons, microglia, and oligodendrocytes were isolated using FANS from unfixed mouse cortices, as described above. Nuclei were pelleted at 500×*g* for 5 min, and supernatant volume was reduced to ∼200–300 μL. Nuclei from each cell type were individually loaded onto an ImageStream Mk II Imaging Flow Cytometer. Brightfield images were captured of each individual nucleus in the sample and the fluorescent signal from the cell-type-specific antibodies used for FANS was measured. IDEAS 6.2 software was used to quantify nuclei size. The intensity of the fluorescent channel (varying by cell type analyzed) was plotted against the aspect ratio of the brightfield channel. Doublets and beads were excluded from downstream analysis. A histogram of gradient root-mean-square (RMS) was plotted to select the most focused brightfield images. A mask was created for the brightfield channel by using the “AdaptiveErode” function. After the mask was applied, area values for nuclei of the three different cell types were exported for statistical analysis.

### Statistics

Analysis of variance (ANOVA) and simple linear regression tests were performed using GraphPad Prism 9 (GraphPad Software, San Diego, California, USA). A posthoc multiple comparison test (Tukey) was used to determine the statistical difference between conditions for ANOVA tests. Statistical significance on graphs is denoted by *p < 0.05, **p < 0.01, and ***p < 0.001. PANTHER Statistical overrepresentation test of gene ontology (GO) terms was performed in PantherDB, by Fisher’s Test with FDR correction to generate p values.^30^

To generate principal component analysis (PCA) plots, all cell type data were processed in the same Progenesis session to use the integral match-between-runs and normalization. PCA plots were generated in R (version 4.0.2) using RStudio (RStudio 2023.06.0 + 421) and the prcomp function with scaling. Ellipses for annotation were generated using the ellipse package.

## Results

**Figure 1:**
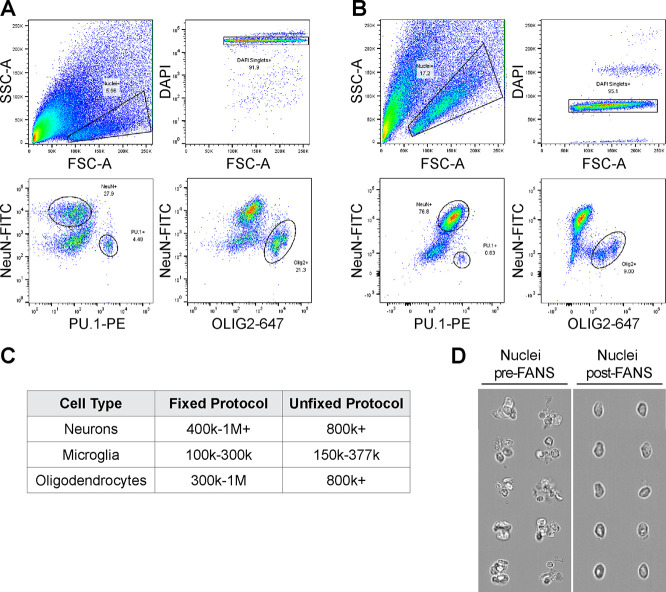
Nuclear isolation from unfixed brain tissue for mass spectrometry compared to fixed samples. (A) FANS plot depicting isolation of neurons, microglia, and oligodendrocytes from unfixed mouse brain. (B) FANS plot depicting isolation of neurons, microglia, and oligodendrocytes from fixed tissue. (C) Average number of nuclei for neurons, microglia, and oligodendrocytes isolated from one mouse cortex using unfixed or fixed samples. (D) Representative images of samples pre- and post-FANS using ImageStream showing removal of debris after sorting.

To characterize the nuclear proteome of brain cell types, unfixed nuclei were isolated from the whole mouse brain after the removal of the cerebellum. Optimization of the fluorescence-activated nuclei sorting (FANS) strategy^27^ for mass spectrometry included the addition of a protease inhibitor cocktail throughout the isolation and staining steps, and the removal of bovine serum albumin during nuclei collection (see Methods). Nuclei were isolated from neurons, microglia, and oligodendrocytes following immunostaining for the cell-type nuclear markers NeuN, PU.1, and Olig2, respectively (Figure 1A). Immunostaining of unfixed nuclei for neurons, microglia, and oligodendrocytes generated comparable FANS plots to our established fixed nuclei isolation protocol (Figure 1B).^28^ Higher yields of unfixed nuclei were achievable by excluding the use of density gradients to remove debris. The yield of unfixed cell-type nuclei isolated from one mouse cortex was comparable to or greater than that of nuclei obtained from fixed sample extraction with a density gradient (Figure 1C). Debris was identified and excluded during FANS, and the majority of sorted events were high-quality intact unfixed nuclei, as confirmed by brightfield imaging using an imaging flow cytometer (Figure 1D).

**Figure 2:**
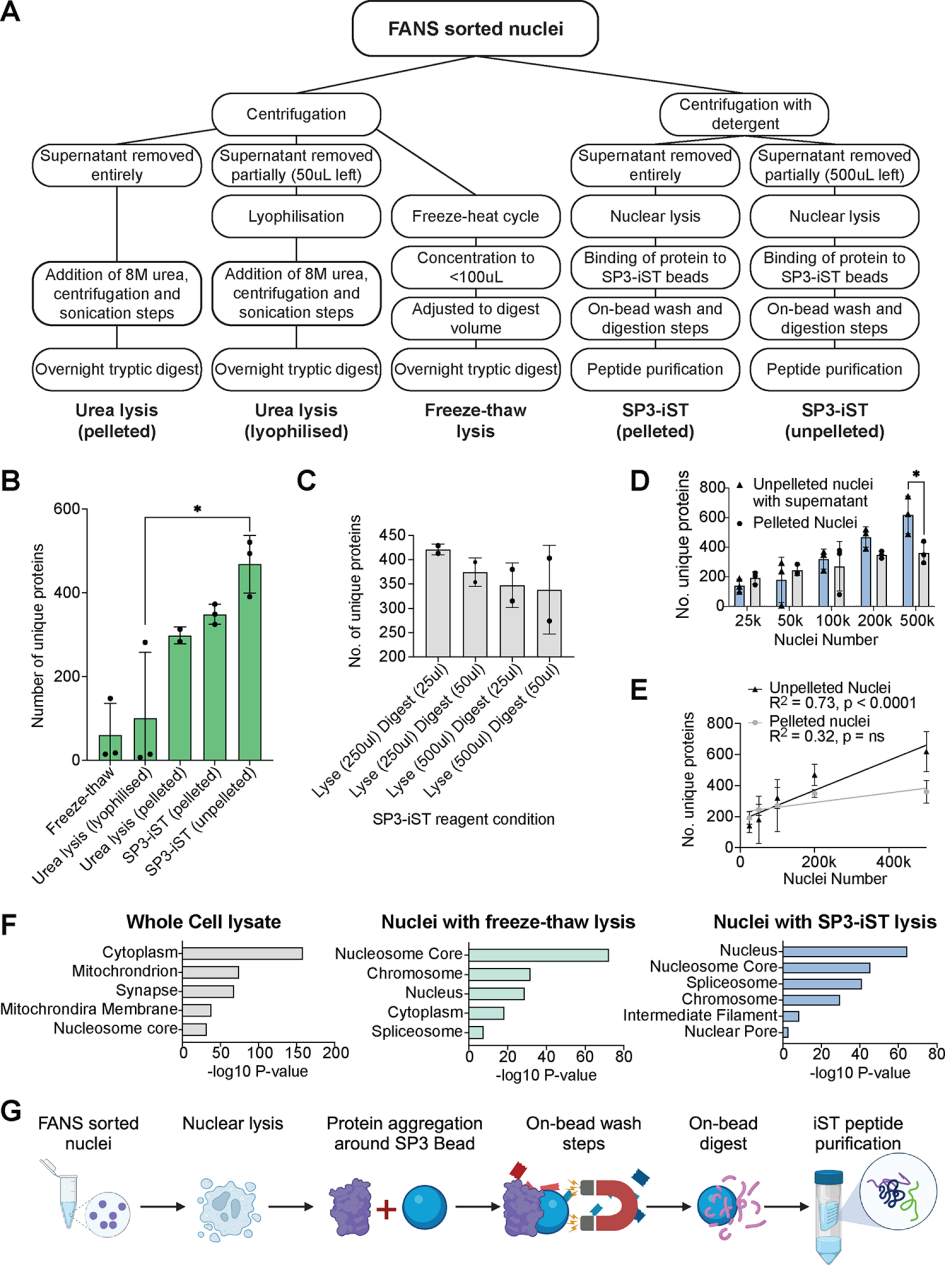
Comparison of nuclei sample preparation for mass spectrometry. (A) Overview of five nuclei sample preparation approaches tested for mass spectrometry: (1) urea lysis–pelleted, (2) urea lysis–lyophilized, (3) freeze–thaw lysis, (4) SP3-iST lysis–pelleted and (5) SP3-iST – nonpelleted. (B) Number of unique proteins identified for each sample preparation approach using 200,000 mouse brain nuclei. *N* = 2, urea (pelleted), *N* = 3, for all other conditions. (C) Number of unique proteins identified with different SP3-iST lyse and digest volumes using 200,000 mouse brain nuclei. *N* = 2 per condition. (D) Serial dilution experiments, showing protein yield for 25,000, 50,000, 100,000, 200,000 and 500,000 mouse brain nuclei using the SP3-iST – pelleted and SP3-iST – nonpelleted sample preparation approaches. *N* = 3 per condition. (E) Linear regression of unique protein numbers identified with 25,000, 50,000, 100,000, 200,000 and 500,000 mouse brain nuclei prepared using SP3-iST – pelleted (black) and SP3-iST – nonpelleted (gray) sample preparations. Data taken from (D); *N* = 3 per condition. (F) PANTHER gene ontology analysis of cellular ontological categories for proteins identified using mouse brain whole cell lysates, nuclei followed by freeze–thaw lysis and nuclei followed by SP3-iST lysis. (G) Schematic of optimized SP3-iST mass spectrometry sample preparation protocol. Error bars; standard deviation.

Microglia are a relatively rare cell type in the brain, and on average, ∼ 200,000 microglial nuclei were isolated from two mouse cortical hemispheres (Figure 1C). Nuclei lysis methods compatible with mass spectrometry analysis were tested for a low number of nuclei that were collected into relatively large volumes (1.5–2 mL), which yielded low peptide concentrations (<0.05 μg μL^–1^). Pelleting unfixed nuclei requires low centrifugation speeds to ensure that the nuclei remain intact. Approaches to increase the yield of pelleted nuclei post-FANS include the addition of bovine serum albumin (BSA) and mild detergents, which are not compatible with mass spectrometry. Concentrating samples by direct precipitation or spin-filters may result in the loss of hydrophobic proteins, and since proteins are limiting in these samples, we explored alternative methods. Five protocols were compared that combined nuclei lysis, digestion, and peptide recovery. These protocols were (1) urea lysis of pelleted nuclei, (2) urea lysis of lyophilized nuclei, (3) freeze–thaw lysis, (4) SP3-iST lysis of pelleted nuclei, and (5) SP3-iST lysis of unpelleted nuclei (Figure 2A).

Protein preparation methods were tested on 200,000 mouse brain nuclei. The total number of proteins identified was ∼2-fold higher for the iST-SP3 unpelleted method compared to the other protein preparation protocols tested (Figure 2B, Supplemental Table 1). To further optimize the iST-SP3 protocol for low-concentration samples in large volumes, different digest volumes (25 μL or 50 μL) or LYSE (2-FOLD) buffer volumes (250 or 500 μL) were tested, and the protein yields for 200,000 nuclei were compared (Figure 2C). The highest number of proteins were identified using the 250 μL LYSE and 25 μL digest volumes (Figure 2C). These LYSE and digest volumes were used for all subsequent SP3-iST experiments. To determine the minimum number of nuclei required for analysis, mass spectrometry was performed on 25,000–500,000 FANS-isolated nuclei using the SP3-iST protocol, with and without nuclei pelleting (Figure 2D, Supplemental Table 1). There was a linear increase in the number of identified proteins with the number of unpelleted nuclei (Figure 2D, E, Supplemental Table 1). The number of detected proteins had not plateaued at 500,000 unpelleted nuclei, indicating that higher nuclei numbers could be used to detect further proteins. The number of identified proteins using pelleted nuclei was inconsistent and lower compared to unpelleted samples and may reflect inconsistencies in nuclei pelleting efficiency (Figure 2D, E, Supplemental Table 1). Gene ontology analysis of proteins identified using the unpelleted SP3-iST and the freeze–thaw preparation methods identified ontology categories associated with nuclear terms (nucleosome core, chromosome, spliceosome, and nucleus) (Figure 2F). In contrast, extranuclear gene ontology terms (cytoplasm, synapse, and mitochondrion) were identified using proteins identified from whole mouse brain lysates without nuclei enrichment (Figure 2F). Based on these findings, subsequent cell type analysis of the nuclear proteome was examined using the SP3-iST lysis of unpelleted nuclei protocol (Figure 2G).

**Figure 3:**
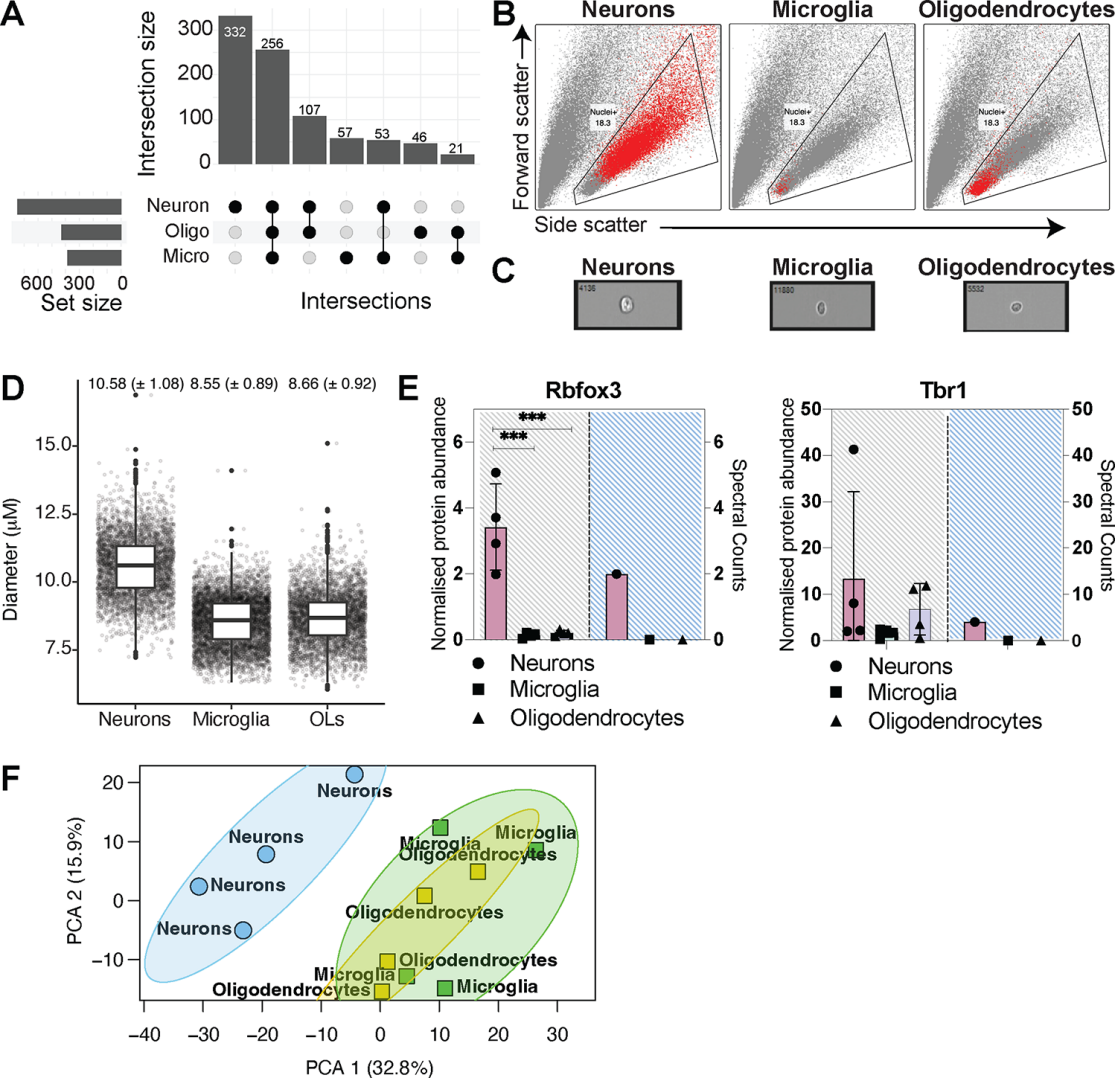
Nuclear proteomes of the major mouse brain cell types. (A) Number of unique and overlapping proteins identified in mouse cortical neurons, microglia and oligodendrocytes (*N* = 4 biological replicates per cell type). (B) Representative forward vs side scatter FANS dot plots showing celltype-specific variation in nuclei size. Nuclei are highlighted in red according to cell type of origin as annotated. (C) Representative images of nuclei from neurons, microglia and oligodendrocytes captured using ImageStream. (D) Nuclei diameter (μm^2^) for neurons, microglia and oligodendrocytes, mean ±1 standard deviation is indicated (*N* = 4,046, 6250, 4386 for neuronal, microglial and oligodendrocyte nuclei, respectively, from a single experiment). (E) Normalized protein abundance and spectral counts for Rbfox3 and Tbr1 for neurons, microglia and oligodendrocytes. One-way ANOVA, Tukey’s multiple testing comparison. Error bars; standard deviation. (F) PCA plot generated using nuclear proteins identified in neurons, microglia and oligodendrocytes (*N* = 4 biological replicates per cell type).

Cell type-enriched nuclear proteomic data for neurons, microglia, and oligodendrocytes was generated by coupling fluorescence-activated nuclei sorting with the SP3-iST lysis of unpelleted nuclei. Among these cell types, microglia are the least abundant in the brain. Approximately 200,000 PU.1^+ve^ microglia nuclei could be routinely isolated per whole mouse brain, excluding the cerebellum (Figure 1C). To provide consistency between cell types, 200,000 nuclei were isolated for each cell type (neurons, microglia, and oligodendrocytes) and processed for downstream mass spectrometry analysis. The total number of proteins identified for neurons was 748, which was >1.7-fold higher than the number of proteins identified for microglia (387 proteins) and oligodendrocytes (430 proteins) (Figure 3A, Supplemental Table 2). The higher number of nuclear proteins identified for neurons compared to the other cell types may reflect differences in nuclei size (as the number of nuclei was the same). Neuronal nuclei were found to be larger compared to microglial and oligodendrocyte nuclei when comparing side scatter and forward scatter profiles following FANS (Figure 3B). Nuclei size was quantified by imaging each nucleus in-stream using an ImageStream^X^ Mark II Imaging Flow Cytometer (Figure 3C). Neuronal nuclei had a mean diameter of 10.58 μm, whereas microglia and oligodendrocyte nuclei had smaller mean diameters of 8.55 and 8.66 μm, respectively (Figure 3D). While we identify several oligodendrocyte and microglia-specific proteins, we did not identify Olig2 or PU.1, most likely due to their relative low abundance in comparison to the identified proteins. We have identified two neuronal-specific markers, Neu1 (*Rbfox1*) and Tbr1 (Table 1) that had spectral matches only in neuronal samples (Supplementary Table 2). Match-between-runs (MBR) identified low levels of Rbfox1 precursor in microglia and oligodendrocytes, though in the order of 15–30x less (Figure 3E). MBR suggested that Tbr1 was similarly expressed in both neurons and oligodendrocytes (though spectral counts were only present for neuronal samples), but much lower in microglia (Figure 3E). Tbr1 is known for its role in neuronal differentiation and migration in the cortex.^31^ However, overexpression of Tbr1 in adult olfactory bulb stem cells increases the production of both neurons and oligodendrocytes while inhibiting astrocytes.^32^

**Table 1:** Select Nuclear Proteins Identified in Neurons, Microglia, and Oligodendrocytes^a^

neurons		microglia	
**transcriptional regulation**			
Actl6b	Baf53b; actin like 6B	Creb1	CAMP responsive element binding protein 1
Ahdc1	AT-hook DNA binding motif containing 1	Psip1	PC4 and SRSF1 interacting protein 1
Bd11a	BCL11 transcription factor A	Sap18	Sin3A associated protein 18
Chas	chromodomain helicase DNA binding protein 5	Supt5h	SPT5 homologue, DSIF elongation factor subunit
Hdac2	histone deacetylase 2	Tbl1xr1	TBL1X/Y related 1
Scrt1	scratch family transcriptional repressor 1	Tef20	transcription factor 20
Sin3a	SIN3 transcription regulator family member A	Znf280d	zinc finger protein 280D
Smarca4	SWI/SNF related, matrix associated, actin dependent regulator of chromatin, subfamily A, member 4		
Tbr1	T-box brain transcription factor 1		
Top2a	DNA topoisomerase II alpha		
**RNA binding proteins**			
Celf2	CUGBP Elav-like family member 2	Fip1l1	factor interacting with PAPOLA and CPSF1
Elavl2	ELAV like RNA binding protein 2	Hnrnpc	heterogeneous nuclear ribonucleoprotein C
Hnrnpl	heterogeneous nuclear ribonucleoprotein L	Khdrbs3	KH RNA binding domain containing, signal transduction associated 3
Prpf40b	pre-MRNA processing factor 40 homologue B	Raly	RALY heterogeneous nuclear ribonucleprotein
Rbfox1	RNA binding fox-1 homologue 1	sf3b6	splicing factor 3b subunit 6
Tardbp	TDP-43; TAR DNA binding protein	Utp18	UTP18 small subunit processome component
**kinases and regulators**			
Akap8l	A-kinase anchoring protein 8 like	Alaps	A-kinase anchoring protein 5
Camk2b	calcium/calmodulin dependent protein kinase II beta	Blk	BLX proto-oncogene, Src family tyrosine kinase
Cdk13	cyclin dependent kinase 13	Fyb1	FYN binding protein 1
**nuclear structure and organization**			
Kpna1	karyopherin subunit alpha 1	Lmnb1	lamin B1
sun1	Sad1 and UNC84 domain containing 1	Smc1a	structural maintenance of chromosomes 1A
**ubiqiutin signaling and DNA damage**			
Brcc3	BRCA1/BRCA2-containing complex subunit 3		
Nosip	nitric oxide synthase interacting protein		
Uchl1	ubiquitin C-terminal hydrolase L1		
Usp39	ubiquitin specific peptidase 39		

oligodendrocytes		common	
**transcriptional regulation**			
Bclaf1	BCL2 associated transcription factor 1	Ints1	integrator complex subunit 1
Chtop	chromatin target of PRMT1	Hdac1	histone deacetylase 1
Ing3	inhibitor of growth family member 3	Nr3C1	nuclear receptor subfamily 3 group C member 1
Ruvbl2	RuvB like AAA ATPase 2	Mecp2	methyl-CpG binding protein 2
Smarca2	SWI/SNF related, matrix associated, actin dependent regulator of chromatin, subfamily A, member 2	Parp1	poly(ADP-ribose) polymerase 1
		Polr2a	RNA polymerase II subunit A
		Smarcd2	SWI/SNF related, matrix associated, actin dependent regulator of chromatin, subfamily D, member 2
		Smarce1	SWI/SNF related, matrix associated, actin dependent regulator of chromatin, subfamily E, member 1
		Top1	DNA topoisomerase I
		Tco2b	DNA topoisomerase II beta
**RNA binding proteins**			
Ddx5	DEAD-box helicase 5	Cirbp	cold inducible RNA binding protein
Elavl3	ELAV like RNA binding protein 3	Elavl1	ELAV like RNA binding protein 1
Hnrnpdl	heterogeneous nuclear ribonucleoprotein D like	Fus	FUS RNA binding protein
Pcbp1	poly(RC) binding protein 1		
Rbm15	RNA binding motif protein 15		
Srsf6	serine and arginine rich splicing factor 6		
**kinases and regulators**			
Ywhaq	tyrosine 3-monooxygenase/tryptophan 5-monooxygenase activation protein theta	Csnk2a1	casein kinase 2 alpha 1
		Pip4k2b	phosphatidylinasitol-5-phasphate 4-kinase type 2 beta
		Srpk1	SRSF protein kinase 1
**nuclear structure and organization**			
Kpna3	karyopherin suburit alpha 3	Kpnb1	karyopherin subunit beta 1
Kpna6	karyopherin subunit alpha 6	Lbr	lamin B receptor
		Nup160	nucleoporin 160
		Tmpo	thymopoietin
**ubiqiutin signaling and DNA damage**			
Ddb1	damage specific DNA binding protein 1	Fau	FAU ubiquitin like and ribosomal protein S30 fusion
Rad21	RAD21 cohesin complex component	Prpf19	pre-MRNA processing factor 19
		Sumo1	small ubiquitin like modifier 1
		Ubc	ubiquitin C

a Select nuclear proteins associated with transcriptional regulation, RNA binding, kinase activity, nuclear structure and organization, and ubiquitin signaling and DNA damage. Proteins were allocated according to cell type enrichment (neurons, microglia, and oligodendrocytes) and identification across all three cell types (common).

Principle component analysis of nuclear proteins common to all cell types showed that neuronal nuclei clustered distinctly from microglia and oligodendrocytes. This likely reflects the higher protein concentrations from neuronal samples (arising from having larger nuclei) compared to microglia and oligodendrocytes (Figure 3F). However, nuclear proteins were identified as unique or commonly shared by each cell type (Figure 3A, Supplemental Table 2). As expected, proteins identified in the nucleus were associated with transcriptional regulation, RNA binding, kinase activity, nuclear structure and organization, ubiquitin signaling, and DNA damage (Table 1). Proteomics of neuronal nuclei identified chromatin regulators known to be enriched in neurons and important for neurodevelopment, such as Hdac2, Chd5, and Tbr1^33−36,31^ (Table 1). In microglia, we identified Fyb1, which is a binding protein of Fyn and a modulator of IL-2 expression, and has been implicated in microglial homeostasis^37^ (Table 1). Nuclear proteins were identified across all three cell types, including proteins broadly implicated in transcription (Polr2a and Parp1), chromatin topology (Top2b and Top1), and nuclear structure (Lbr and Nup160) (Table 1). Proteins identified in both neurons and glia were also associated with transcriptional regulation (Hdac1, Smarcd2, Smarce1) and RNA processing (Fus)^38,39,33,40^ (Table 1).

## Discussion

This study presents a method for analyzing nuclear-specific proteins from FANS-sorted nuclei isolated from frozen tissue. One of the challenges was overcoming relatively large volumes of sorting buffer without compromising protein extraction and digestion efficiency. Pelleting the unfixed nuclei required the addition of detergents or additional proteins to minimize loss of the nuclei when aspirating the supernatant which can complicate mass spectrometry sample preparation and downstream analysis.

We integrated the processes of nuclear lysis and protein extraction with Solid-Phase-Enhanced Sample Preparation (SP3), as described by.^41^ This approach not only reduced the need for preconcentrating the nuclei through high-speed centrifugation but also facilitated the removal of detergents present in low concentrations. Our experiments demonstrated that, while it was possible to pellet the nuclei at 600×*g* using 0.5% (v/v) Triton, the resulting nuclei pellet was relatively fragile. Consequently, attempting to remove the supernatant often led to a loss of nuclei and we did not observe a significant increase in the number of proteins detected when comparing samples with 25,000 nuclei to those with 500,000 nuclei (Figure 2C). However, retaining 500 μL of the supernatant after centrifugation and optimizing the proportions of SP3-iST reagents accordingly allowed for much-improved retention of the nuclei. The number of proteins identified scaled with the number of nuclei, enabling protein identifications from samples as low as 25,000 nuclei.

The number of proteins identified was relatively modest compared to other nuclear-specific studies (e.g., > 4000 proteins identified by^42^). It is likely that we have only analyzed an abundant subset of the available nuclear proteome. We attribute this to an increase in the purity of our nuclear isolation by FANS (in comparison to centrifugation-based approaches) meaning there is low non-nuclear/cytosolic protein carry-over and a limited starting amount of protein. Dammer et al. have also demonstrated the feasibility of extracting nuclei from unfixed post-mortem brain tissue.^43^ They isolated 5 million nuclei by FANS, reporting a high degree of enrichment for nuclear proteins.^43^ In line with our observations, they noted that lower yields of protein than expected was obtained, likely due to an enhanced degree of nuclear-purity from FANS. While we isolate fewer nuclei in this study (200,000 versus 5,000,000), with fewer protein identifications (952 versus 1755), there is some overlap in the mouse/human-equivalent neuron-specific RNA-binding proteins, transcription factors and protein kinases from both studies. In this study, we extended and optimized this approach for the isolation of rare cell-type populations, minimizing postsorting centrifugation to aid protein recovery. While we have implemented this method in mouse brain, we believe the isolation and sample preparation would be directly transferable to brain tissue from other organisms, including humans, similar to as described in Dammer et al. Among the most abundant proteins identified were histone proteins H2A/B, H3, and H4. Future iterations of our approach could incorporate a histone precipitation step to either enrich histones for the assessment of posttranslational modifications or to deplete histones for the enrichment of low-abundance chromatin regulators. In addition, our approach can be used to assess the post-translational modification of chromatin regulators, which can alter their function. For example, the activity of Sirtuin2 (SIRT2) can be inhibited by phosphorylation of serine 331 and is increased in the nucleus of AD neurons.^44^

Our method for analyzing FANS-sorted nuclear-specific proteins was applied to three major cell types of the mouse brain. Our approach identified nuclear proteins associated with gene regulatory functions, many of which have genetic links with brain-related conditions. Our neuronal nuclei proteomics identified chromatin regulators implicated in neurodevelopmental conditions, such as TBR1 syndrome (or intellectual development disorder with autism and speech (IDDAS) delay) and amyotrophic lateral sclerosis (ALS)/frontotemporal lobar degeneration (FTLD) (TDP-43, protein product of *Tardbp*)^45,46^ (Table 1). In microglia, we detected Tbl1xr1, which is also expressed outside the brain and has been linked to developmental phenotypes, including West syndrome, Pierpont syndrome, autism spectrum conditions, and microcephaly among others.^47−50^ Subunits of the SWI/SNF chromatin remodeling BAF complex were identified across all three cell types (Smarca2, Smarca4, Smarcd2, Smarce1), and are a family of factors implicated in neurodevelopmental disorders.^51^ Likewise, nuclear proteins identified across all three cell types have been linked to human genetic mutations associated with neurodevelopmental disorders (Mecp2) and neurodegenerative diseases (Fus).^52−54^ In conclusion, our approach allows the detection of subcellular proteins localized to the nucleus from unfixed samples, with known implications in diseases that are expressed in rare brain cell types. Our approach opens new avenues of research into the study of chromatin regulators in the context of brain development, aging, and disease.

## Data Availability

The mass spectrometry proteomics data have been deposited to the ProteomeXchange Consortium via the PRIDE^55^ partner repository with the data set identifier PXD053515.

## Supporting Information Available

Mass spectrometry analysis of nuclei processed using the iST method, freeze-thaw lysis and urea lysis, and mass spectrometry analysis from whole cell lysates and mass spectrometry analysis of brain cell type nuclei for microglia, neurons, and oligodendrocytes (PDF)
Mass spectrometry analysis of nuclei processed using the iST method, freeze thaw lysis and urea lysis, and mass spectrometry analysis from whole cell lysates (XLSX)
Mass spectrometry analysis of brain cell type nuclei for microglia, neurons and oligodendrocytes (XLSX)
