# Supplementary material for: FANS Unfixed: Isolation and Proteomic Analysis of Mouse Cell Type-Specific Brain Nuclei
Source: J Proteome Res. 2024 Jul 26;23(9):3847–57. doi: 10.1021/acs.jproteome.4c00161 (PMC11385383; doi:10.1021/acs.jproteome.4c00161)
Supplement: Supplementary file 1 — pr4c00161_si_001.pdf [file pr4c00161_si_001.pdf]

Supporting Information

FANS unfixed: isolation and proteomic analysis of mouse cell type-specific brain nuclei.

Lucy Bedwell[1,2], Myrto Mavrotas[1], Nikita Demchenko[3], Reuben M. Yaa[1,2], Brittannie Willis[1,4], Zuzana Demianova[5], Nelofer Syed[1], Harry J. Whitwell[4*], Alexi Nott[1,2*]

[1.] Department of Brain Sciences, Imperial College London, London, W12 0NN, UK.
[2.] UK Dementia Research Institute, Imperial College London, London, W12 0NN, UK.
[3.] MRC Laboratory of Medical Sciences, Du Cane Road, London, W12 0NN, UK.
[4.] Department of Metabolism, Digestion, and Reproduction, Imperial College London, London, W12 0NN, UK.
[5.] PreOmics GmbH, Martinsried, D-82152, DE

[*]Co-corresponding authors: h.whitwell@imperial.ac.uk and a.nott@imperial.ac.uk

Table of contents

Table S1 Mass spectrometry analysis of nuclei processed using the iST method, freeze-thaw lysis and urea lysis, and mass spectrometry analysis from whole cell lysates (corresponding to Fig. 1,2)
Table S2 Mass spectrometry analysis of brain cell type nuclei for microglia, neurons and oligodendrocytes (corresponding to Fig. 3).
